# Comparative Studies on the Stenogamous and Eurygamous Behavior of Eight *Anopheles* Species of the Hyrcanus Group (Diptera: Culicidae) in Thailand

**DOI:** 10.3390/insects7020011

**Published:** 2016-03-26

**Authors:** Adulsak Wijit, Kritsana Taai, Watcharatip Dedkhad, Chayanit Hempolchom, Sorawat Thongsahuan, Wichai Srisuka, Yasushi Otsuka, Masako Fukuda, Atiporn Saeung

**Affiliations:** 1 Office of Disease Prevention and Control, 1st, Department of Disease Control, Ministry of Public Health, Chiang Mai 50000, Thailand; adulsakw@gmail.com; 2 Department of Parasitology, Faculty of Medicine, Chiang Mai University, Chiang Mai 50200, Thailand; kritsana_taai@hotmail.com (K.T.); watchratip@gmail.com (W.D.); chayanit.hem@gmail.com (C.H.); 3 Faculty of Veterinary Science (Establishment Project), Prince of Songkla University, Songkhla 90110, Thailand; sorawat_ton@hotmail.com; 4 Entomology Section, Queen Sirikit Botanic Garden, P.O. Box 7, Chiang Mai 50180, Thailand; wsrisuka@gmail.com; 5 Research Center for the Pacific Islands, Kagoshima University, Kagoshima 890-8580, Japan; yotsuka@cpi.kagoshima-u.ac.jp; 6 Division of Life Science Research, Research Promotion Institute, Oita University, Hasama, Oita 879-5593, Japan; mfukuda@oita-u.ac.jp

**Keywords:** *Anopheles peditaeniatus*, self-mating, density resting surface, mating behavior, male genitalia

## Abstract

Establishment of laboratory colony is essential for mosquito-borne-disease research. Mating behavior of stenogamous *Anopheles peditaeniatus* and seven eurygamous species (*Anopheles argyropus*, *Anopheles crawfordi*, *Anopheles nigerrimus*, *Anopheles nitidus*, *Anopheles paraliae* (=*An*. *lesteri*), *Anopheles pursati* and *Anopheles sinensis*), were investigated and compared in this study. The self-mating success of adult mosquitoes in different size cages at two density resting surface (DRS) values, 3.6 and 7.2, was statistically significant between stenogamous and eurygamous species. The results obtained from comparative measurements of specific characters in adult females (maxillary palpomere and antennal sensilla characters) and males (wing and genitalia) indicate those characters might influence the mating success of *An. peditaeniatus* in a small cage. The gonostylus of *An. peditaeniatus* was shorter than the eurygamous species. Additionally, the lower frequency of clasper movement and shorter mating time could be important mechanisms that control the stenogamous behavior of *An. peditaeniatus*. Interestingly, for the first time, a cluster of large sensilla coeloconica was recorded on the antenna of *An. argyropus* and *An. peditaeniatus* females. There was no statistically significant difference in the mean number per female of those large antennal sensilla coeloconica among six of the eurygamous species.

## 1. Introduction

At least 27 species belonging to the Hyrcanus Group (Genus *Anopheles*, Subgenus *Anopheles*) [1] are distributed widely from Europe to East and Southeast Asia, including some of the off-lying islands of the Indian and Pacific Oceans. In Thailand, eight species of the Hyrcanus Group have been reported so far, *i.e.*, *Anopheles argyropus*, *Anopheles crawfordi*, *Anopheles nigerrimus*, *Anopheles nitidus*, *Anopheles paraliae* (=*An*. *lesteri*), *Anopheles peditaeniatus*, *Anopheles pursati* and *Anopheles sinensis* [2,3]. Remarkably, *An*. *paraliae* was reported as synonymous with *An*. *lesteri* (Korea strain) based on low pairwise genetic distance for ITS2, COI and COII sequences and genetic compatibility in crossing-mating experiments [1,4]. Among these, *An. peditaeniatus* and *An. sinensis* are both considered as suspected and natural vectors of *Plasmodium vivax* in Thailand [5,6,7,8,9], and China and Korea, respectively [10,11,12,13,14,15,16]. These species are also natural vectors of Japanese encephalitis virus in China and India [17,18]. *Anopheles sinensis* and *An*. *nigerrimus* have been incriminated as a main vector and secondary or incidental vector, respectively, of *Wuchereria bancrofti* in Asia [19]. In addition, *An. peditaeniatus*, *An. crawfordi*, *An. nigerrimus*, *An. argyropus* and *An. pursati* were reported as high potential vectors of nocturnally subperiodic *Brugia malayi* [20]. The Hyrcanus Group is considered also as an economic pest of cattle because of its vicious biting-behavior and ability to transmit cervid filariae of the genus, *Setaria* [2,21]. Colony establishment is fundamental for mosquito-borne-disease research, and the inability to maintain a healthy colony of difficult-to-rear species is a principal cause of many failed research efforts. In natural conditions, anopheline females are mated when entering swarms of males, which usually appear above tops of bushes and other objects. Each female is caught by a male that locates her from a flight tone, which is proportional to wing size and wing-beat frequency. This flight tone is heard through the hearing organs (Johnston’s organ) in the antennae, and after coupling the two mosquitoes fall out from the swarm [22,23,24,25]. Nonetheless, success in couples catching each other is based entirely on contact with the sex pheromone of conspecific females and males. Furthermore, detection of this active substance involves a number of olfactory receptors (e.g., sensilla trichodea, sensilla basiconica and sensilla coeloconica) located on antennal segments [26]. In laboratory conditions, the limited space in a standard 30 cubic cm cage or other small area appears to inhibit or reduce the formation of dancing male swarms. Therefore, this causes copulation failure (eurygamous behavior), while many species belonging to the genus *Aedes*, *Culex* and *Mansonia* can copulate without male swarms, and mate easily in small spaces (stenogamous behavior) [26,27,28]. In addition, evidence of the difference in male genitalia morphometry, frequency of clasper movements and mating time between stenogamous *Anopheles cracens* and eurygamous *Anopheles dirus* have been documented [29].

More recently, Wijit *et al.* [30] developed the first DNA barcodes for species identification, and screened the stenogamous behavior of the eight Hyrcanus Group species in Thailand. The results revealed that only five species, *i.e.*, *An. pursati*, *An. sinensis*, *An. nigerrimus*, *An. paraliae* and *An.* *peditaeniatus*, could oviposit successfully, with insemination rates of 31%, 33%, 42%, 50% and 77%, respectively. The remaining three species, *An. argyropus*, *An. crawfordi* and *An. nitidus*, failed to copulate in the laboratory cages. After selection, the stenogamous colony of *An.* *peditaeniatus* was maintained as a self-mating colony for more than 20 generations, with insemination rates that ranged from 61%–86%. In contrast, *An. sinensis*, *An. pursati*, *An. nigerrimus* and *An. paraliae* thereafter failed to copulate naturally under the same conditions. Therefore, only a self-mating colony of *An*. *peditaeniatus* was established successfully in their study.

However, there is still a lack of knowledge on the possible mechanism(s) that controls stenogamous and eurygamous behavior of the eight Hyrcanus Group species in this country. Therefore, this study made detailed investigation by comparing: (1) the mating ability of adult mosquitoes in a 10, 20, 30 and 40 cubic cm cage at two density resting surface (DRS) of 3.6 and 7.2; (2) the measurements of male and female wings, female maxillary palpomeres and male genitalia; (3) the number of large sensilla coeloconica on the antennae of females; and (4) the frequency of clasper movement in male genitalia during induced copulation, and duration of mating between stenogamous and eurygamous species.

## 2. Methods

### 2.1. Field Collection and Species Identification of the Hyrcanus Group

Five iso-female lines (isolines) of each eight *Anopheles* species of the Hyrcanus Group were collected in six provinces of western and southern Thailand as previously reported by Wijit *et al.* [30]. The species and strains were as follows: *An. argyropus* (Nakhon Si Thammarat strain: 08°29′ N, 100°0′ E), *An. crawfordi* (Trang strain: 07°33′ N, 99°38′ E), *An. nigerrimus* (Songkhla strain: 07°13′ N, 100°37′ E), *An. nitidus* (Phang Nga strain: 08°27′ N, 98°31′ E), *An. paraliae* (Ratchaburi strain: 13°30′ N, 99°54′ E), *An. peditaeniatus* (Phang Nga strain: 08°27′ N, 98°31′ E), *An. pursati* (Ratchaburi strain: 13°30′ N, 99°54′ E) and *An. sinensis* (Chumphon strain: 10°29′ N, 99°11′ E). Then, they were identified exactly using intact morphology of eggs, larvae, pupal skins and adult females of F_1_-progenies as well as molecular species identification, based on COI barcode sequences (GenBank accession numbers AB781747-AB781786) [30], and maintained further to perform the experiments in this study. Mosquito rearing procedures for the Hyrcanu*s* Group (swamp-breeders) followed the detailed techniques described by Choochote and Saeung [31]. All of the experiments were performed in an insectary at 27 ± 2 °C, 70%–80% relative humidity, and illumination from a combination of natural daylight from a glass window and fluorescent lighting was provided for approximately 12 h a day [30].

### 2.2. Establishment of a Stock Colony

After exact species identification, the stock colonies of eight species of the Hyrcanus Group were established by pooling five iso-female lines of each anopheline species. These stock colonies were used throughout the experiments.

### 2.3. Mating Ability of Adult Mosquitoes in Various Sized Cubic Cages and a DRS

**Figure 1:**
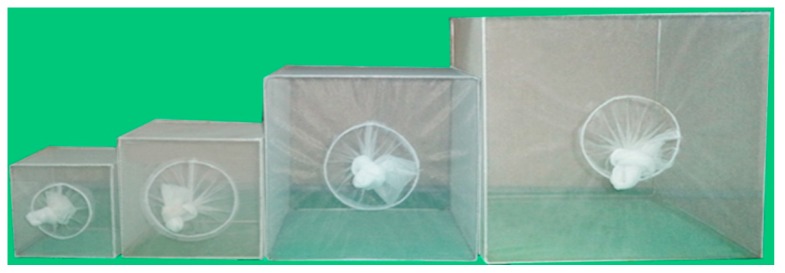
Four cages (left to right: 10, 20, 30 and 40 cubic cm cages) used for self-mating mosquitoes.

Mosquitoes of the 9th generation (F_9_) were used to determine self-mating ability in various sized cubic cages. The reason for using this mosquito generation was based on the fact that any mosquito colony, colonized for more than eight consecutive generations, was of adaptive laboratory mosquito-strains, and easily maintained and mass produced for any experiments [30]. The Density resting surface (DRS) was calculated by dividing the vertical resting surface area (RS) of the cages (cm^2^) by the mosquito population density (D) [32]. Comparing the mating ability of adult mosquitoes in a 10, 20 and 30 cubic cm cage at a DRS of 3.6 and that in a 10, 20, 30 and 40 cubic cm cage at a DRS of 7.2, was carried out using various numbers of female/male mosquitoes (F_9_) (Figure 1). At a DRS of 3.6, the number of females/males (total) 44/67 (111), 178/267 (445) and 400/600 (1000) was introduced into a 10, 20 and 30 cubic cm cage, respectively. However, the 40 cubic cm cage was not used for the DRS of 3.6, since this experiment needs large total numbers of adult mosquitoes (1788). At a DRS of 7.2, the number of females/males (total) 22/34 (56), 89/133 (222), 200/300 (500) and 355/533 (888) was introduced into a 10, 20, 30 and 40 cubic cm cage, respectively, where they co-habited for one week. Then, both 10% sucrose and 5% multivitamin syrup solutions were provided as adult nutrients.

**Figure 2:**
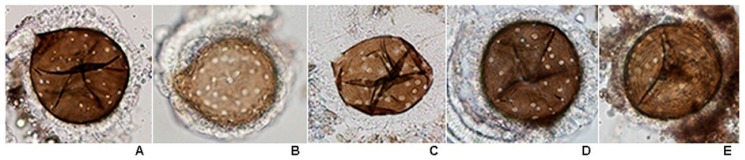
Grading of sperm within spermatheca of inseminated females of the eight species of the Hyrcanus Group: (**A**) 0; (**B**) 1+; (**C**) 2+; (**D**) 3+; and (**E**) 4+ (100× magnification).

The mean insemination rate was calculated from dissection of a total of 200 spermathecae (duplicate experiments, 100 spermathecae/experiment), and examined for evidence of insemination status. Movement of the long thread-like spermatozoa within the spermathecae was looked for under 100× magnification with a compound microscope (Olympus BX53, Olympus Corporation, Tokyo, Japan). The spermatozoa appeared as fine concentric threads within the spermathecae and were often seen rotating as a cluster. After placing in a cover slip, the spermathecae were ruptured, and the surrounding field scanned for the spermatozoa. Then, they were graded as 0 (fairly transparent spermatheca or uninseminated), 1+ (25% of sperm in spermatheca), 2+ (50% of sperm in spermatheca), 3+ (75% of sperm in spermatheca) and 4+ (100% or spermatheca full of sperm) (Figure 2).

### 2.4. Measurements and Large Sensilla Coeloconica Counts under Compound Microscope

**Figure 3:**
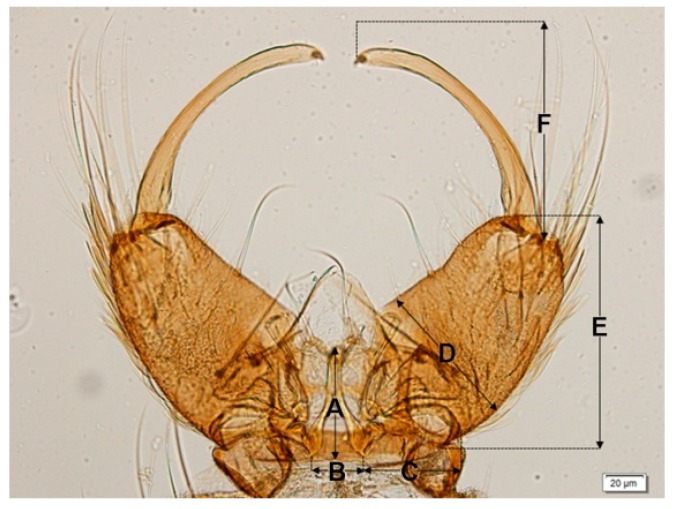
Measurements of the male genitalia (ventral view) at various sites: (**A**) length of the aedeagus; (**B**) width of the aedeagus; (**C**) length between the base of the aedeagus and origin of the gonocoxite; (**D**) width of the gonocoxite at the origin of the parabasal seta; (**E**) Length of the gonocoxite; and (**F**) length of the gonostylus. Scale bar: 0.02 mm.

The measurements of male and female wings, female maxillary palpomeres and male genitalia, and large sensilla coeloconica (=pitted peg) counts of female antennae between stenogamous and eurygamous species were compared by using 36-hour post emerged females and males. Thirty samples from each species were immersed in a small bottle containing 10% potassium hydroxide (KOH) and left for 30–45 min in a hot oven. After clearing, they were washed with 80% ethanol and each structure was dissected using an insect needle. Each sample was slide-mounted with Hoyer’s media. The measurements included: (1) length and width (ratio of length to width) of female and male wings; (2) maxillary palpomere index or palpomere ratio (calculated by dividing the combined length of palpomere 5 and 4 by length of palpomere 3) [33]; and (3) male genitalia (length and width of the aedeagus, length between the base of the aedeagus and origin of the gonocoxite, width of the gonocoxite at the origin of the parabasal seta, length of the gonocoxite and gonostylus) (Figure 3). In addition, the number of large sensilla coeloconica on the flagellum, which was divided into 13 distinct flagellomeres [34], was also counted. All structures were measured and observed under a compound microscope (Olympus BX53).

### 2.5. Frequency of Clasper Movement in Male Genitalia during Induced Copulation, and Mating Times

The frequency of clasper movement in male genitalia during induced copulation, and mating times between stenogamous and eurygamous species were compared by using 5-day-old females and males of each species. During induced copulation, the females were clasped initially with male claspers, and then remained coupled for a long period of time before a pumping motion started, with movement of their claspers (gonocoxite and gonostylus) until separation. The mating time and frequency of clasper movements were measured and counted using an electric watch under a stereoscopic microscope.

### 2.6. Statistical Analysis

The Chi-square test was used to determined insemination rates. Variation observed in anatomical features of adult females and males genitalia, number of large sensilla coeloconica on female antennae, and frequency of clasper movement in male genitalia during induced copulation and mating times was analyzed by one-way analysis of variance (ANOVA). Differences between stenogamous and eurygamous species were compared using Tukey’s HSD (honest significant difference) test. All data were analyzed using SPSS v. 16.0 for Windows (SPSS Inc., Chicago, IL, USA). Statistical significance was set at *p* < 0.05.

## 3. Results

### 3.1. Mating Ability of Adult Mosquitoes in 10, 20 and 30 cubic cm Cage at DRS of 3.6

The comparative mean insemination rates between stenogamous and eurygamous species demonstrated a statistically significant difference (*p* < 0.05) for all cage sizes (Table 1). However, *An.* *peditaeniatus*, which had a high insemination rate, showed no statistically significant difference among cage sizes (*p* > 0.05). Sperm density in the spermathecae of inseminated female mosquitoes of the eight species was based on grading, using various cage sizes at a DRS of 3.6, which are shown in Supplementary Material (Tables S1–S3). More than 50% of inseminated-female *An. peditaeniatus* showed high sperm density (3+ and 4+) in spermathecae in all cage sizes. Low sperm density (1+) was observed mostly in spermathecae of *An*. *argyropus*, *An*. *crawfordi*, *An*. *nigerrimus*, *An*. *nitidus*, *An*. *paraliae* and *An*. *pursati*. The comparative frequency of sperm density, which was high at 3+ and 4+ between *An*. *peditaeniatus* and the four species, demonstrated a statistically significant difference (*p* < 0.05).

### 3.2. Mating Ability of Adult Mosquitoes in 10, 20, 30 and 40 cubic cm Cage at Density Resting Surface (DRS) of 7.2

The comparative mean insemination rates between stenogamous and eurygamous species demonstrated a statistically significant difference (*p* < 0.05) for all cage sizes (Table 2). Nonetheless, *An.* *peditaeniatus*, which had a high insemination rate, showed no statistically significant difference among cage sizes (*p* > 0.05). Sperm density in the spermathecae of inseminated female mosquitoes of the eight species was based on grading, using various cage sizes at a DRS of 7.2, which are shown in Supplementary Material (Tables S4–S7). More than 50% of inseminated-female *An. peditaeniatus* showed high sperm density (3+ and 4+) in spermathecae in all cage sizes. Low sperm density (1+) was observed in the other seven species. The comparative frequency of sperm density, which was high at 3+ and 4+ between *An*. *peditaeniatus* and the four or five species (at 20 cubic cm cage), demonstrated a statistically significant difference (*p* < 0.05).

### 3.3. Measurements of Wings in Adult Females and Males

**Figure 4:**
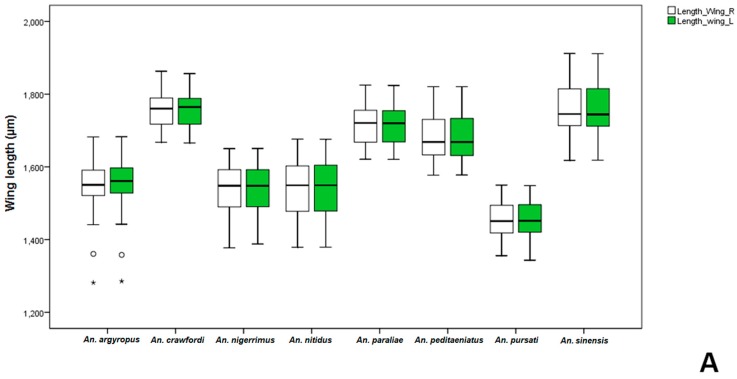

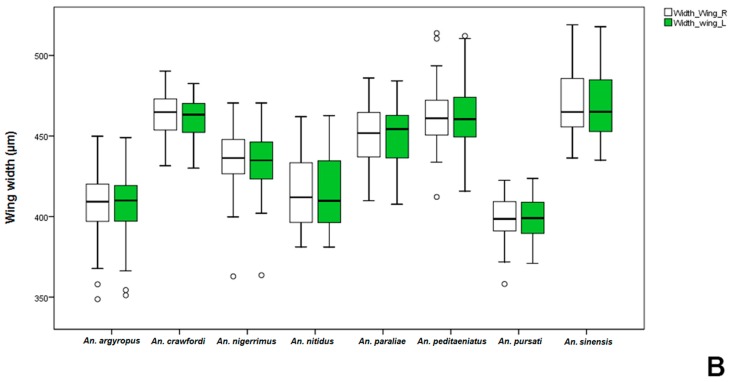
The Box-and-Whisker Plots representation of variation in wing length (**A**) and width (**B**) of adult female of the eight Hyrcanus Group species.

**Figure 5:**
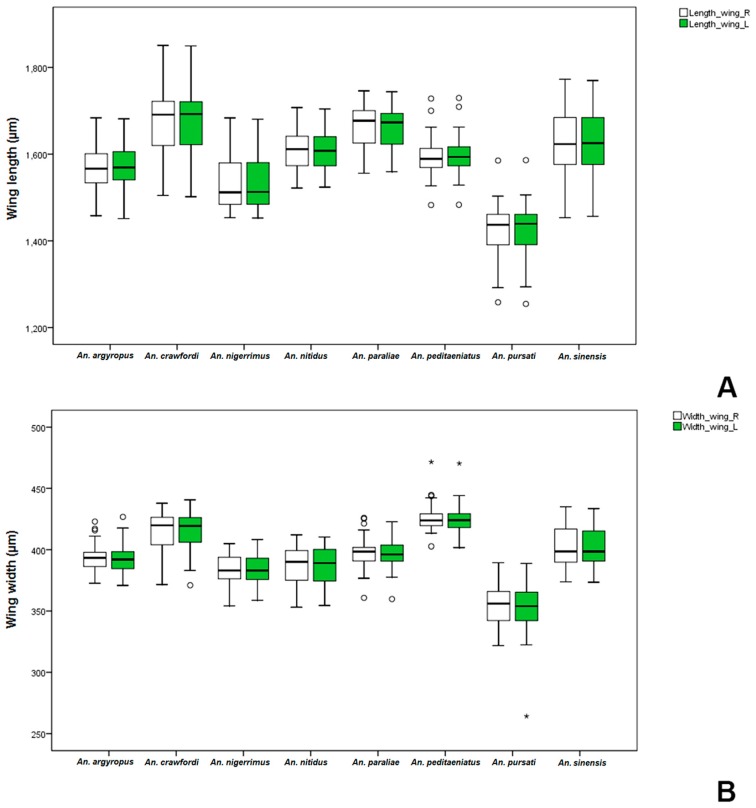
The Box-and-Whisker Plots representation of variation in wing length (**A**) and width (**B**) of adult male of the eight Hyrcanus Group species.

The length and width of wings in females and males of the eight species of the Hyrcanus Group were measured in order to compute a ratio (length/width). Comparisons of mean wing ratios of females of the stenogamous *An. peditaeniatus* and the seven eurygamous species were statistically significant difference (*p* < 0.05), except for *An*. *nigerrimus*, *An. nitidus* and *An. pursati* (*p* > 0.05). Additionally, comparative mean wing ratios of males of the eight species were statistically significant difference (*p* < 0.05) (Table 3). The Box-and-Whisker Plots revealed that *An*. *sinensis* females were longer and wider (Figure 4), whereas, male *An*. *crawfordi* and *An*. *peditaeniatus* were longer and wider, respectively, than other species (Figure 5).

### 3.4. Measurements of Maxillary Palpomeres of Females

**Figure 6:**
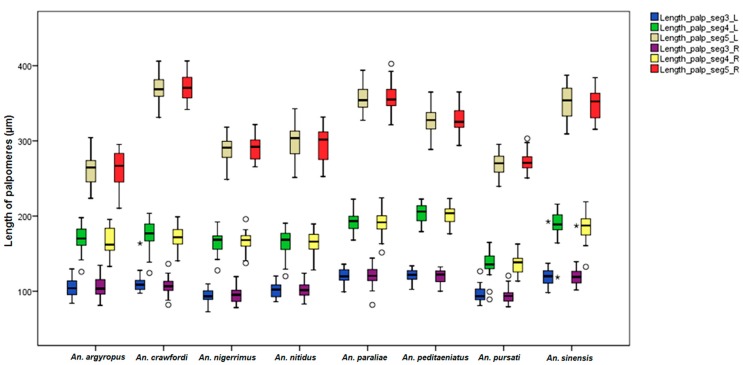
The Box-and-Whisker Plots representation of variation in maxillary palpomeres length of adult female of the eight Hyrcanus Group species.

The results of comparisons of mean palpomeres ratios in females of the stenogamous *An. peditaeniatus* and the seven eurygamous species were statistically significant difference (*p* < 0.05) (Table 4). The Box-and-Whisker Plots revealed that *An*. *paraliae*, *An*. *peditaeniatus* and *An*. *crawfordi* were longer in palpomeres 3, 4 and 5, respectively, than other species (Figure 6).

### 3.5. Number of Large Sensilla Coeloconica on Antennae of Females

**Figure 7:**
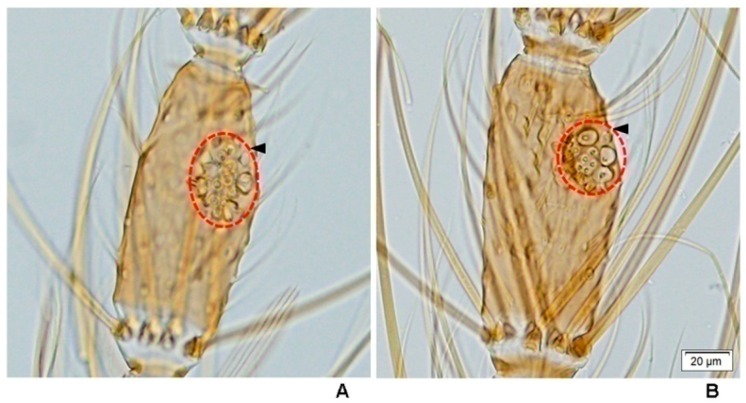
The cluster of large sensilla coeloconica located in sunken depression (sacculus) on the 5th antennal flagellomere of *An. argyropus* (**A**) and *An. peditaeniatus* (**B**), photographed under a compound microscope. Scale bar: 0.02 mm.

**Figure 8:**
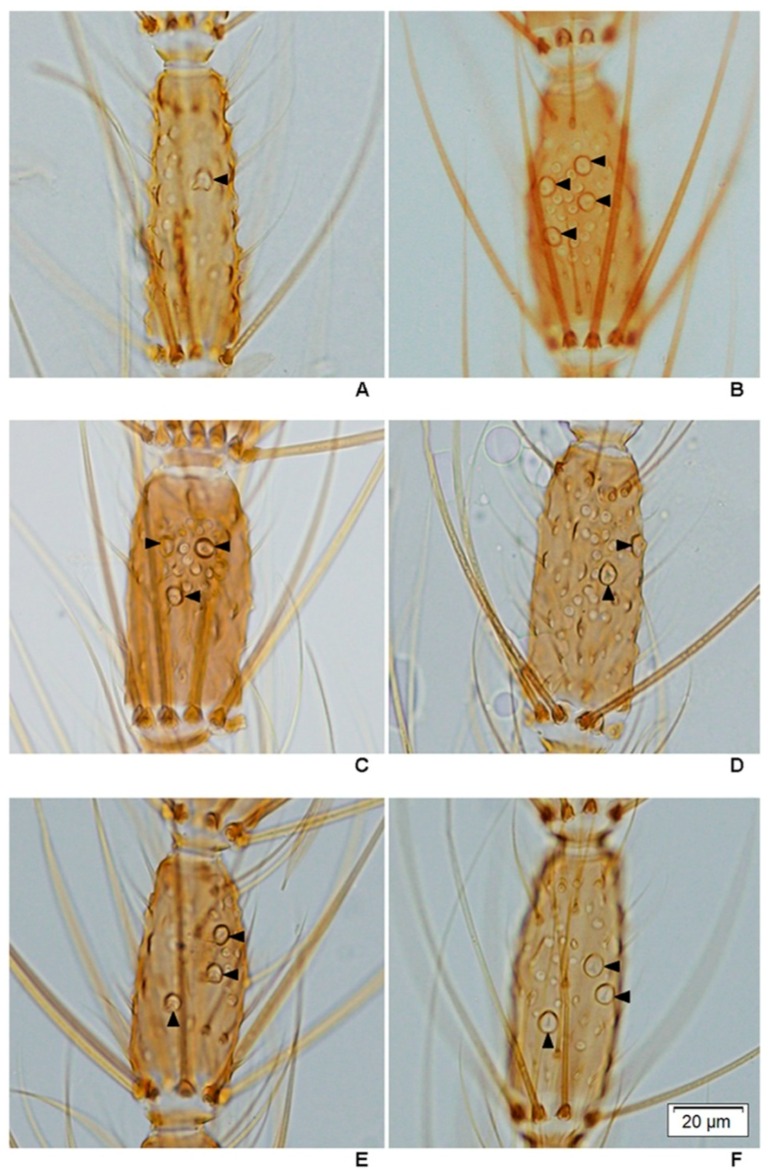
The distribution of large sensilla coeloconica on the 5th antennal flagellomere of six species of the Hyrcanus Group, photographed under a compound microscope: (**A**) *An. crawfordi*; (**B**) *An. nitidus*; (**C**) *An. nigerrimus*; (**D**) *An. paraliae*; (**E**) *An. pursati*; and (**F**) *An. sinensis*. Scale bar: 0.02 mm.

Attempts were made to count the number of large sensilla coeloconica on the antennal flagellum of females of the eight species of the Hyrcanus Group. However, it was not possible to count individual sensilla of *An. argyropus* and *An. peditaeniatus* as they are arrayed in a cluster on the flagellomeres (Figure 7). The cluster of large sensilla coeloconica occurs mainly on flagellomeres 3–7 of *An. argyropus* and *An*. *peditaeniatus*. However, the large sensilla coeloconica of *An*. *peditaeniatus* are located as a cluster in deeper sacculi than in *An*. *argyropus*. In contrast, the other six species have sensilla that occur as a single pit on their flagellomeres (Figure 8). The mean number of large sensilla coeloconica on the antennal flagellum of females was calculated from 30 individuals of each of the six species; the means are shown in Table 5. The number of large sensilla coeloconica per antennal flagellomere ranged from 0–6, 0–7, 0–6, 0–9, 0–6 and 0–8 in *An*. *crawfordi*, *An*. *nigerrimus*, *An*. *nitidus*, *An*. *paraliae*, *An*. *pursati* and *An*. *sinensis*, respectively. The total number of large sensilla coeloconica per antennae ranged between 45–63, 56–78, 50–69, 52–89, 51–69 and 57–88 in *An*. *crawfordi*, *An*. *nigerrimus*, *An*. *nitidus*, *An*. *paraliae*, *An*. *pursati* and *An*. *sinensis*, respectively. The mean numbers of large sensilla coeloconica per antennae of the six species were not statistically significant difference (*p* > 0.05). It is interesting to note that no large sensilla coeloconica are present on the terminal flagellomere (13) of *An*. *nitidus* (Table 5).

### 3.6. Measurements of Male Genitalia 

**Figure 9:**
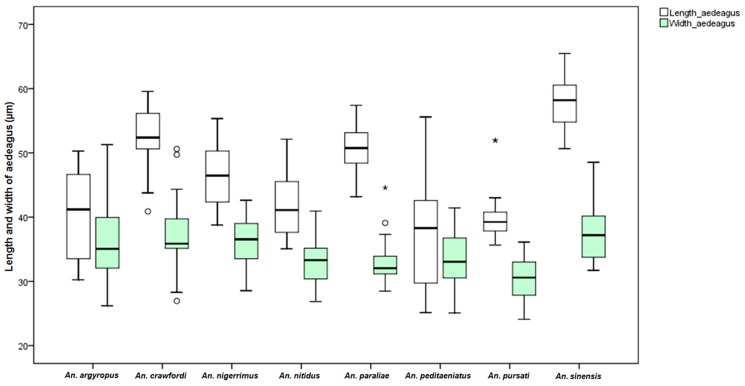
The Box-and-Whisker Plots representation of variation in aedeagus length and width of adult male of the eight Hyrcanus Group species.

**Figure 10:**
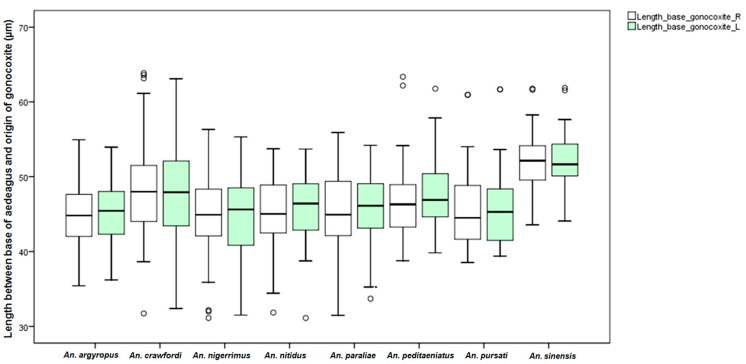
The Box-and-Whisker Plots representation of variation in length between base of aedeagus and origin of gonocoxite of adult male of the eight Hyrcanus Group species.

**Figure 11:**
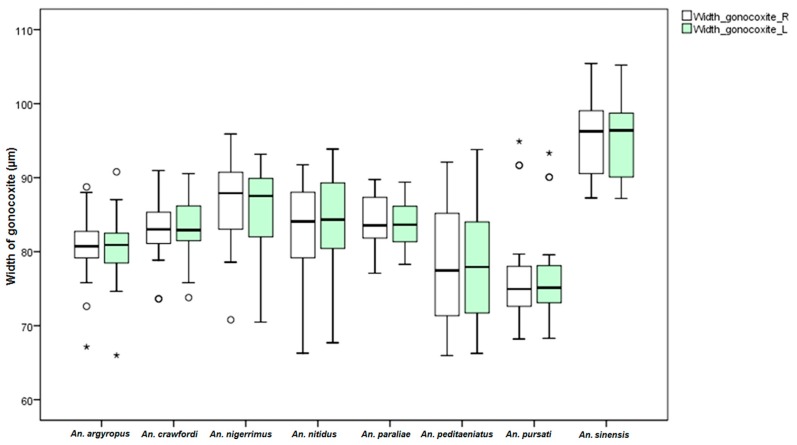
The Box-and-Whisker Plots representation of variation in width of gonocoxite at origin of parabasal seta of adult male of the eight Hyrcanus Group species.

**Figure 12:**
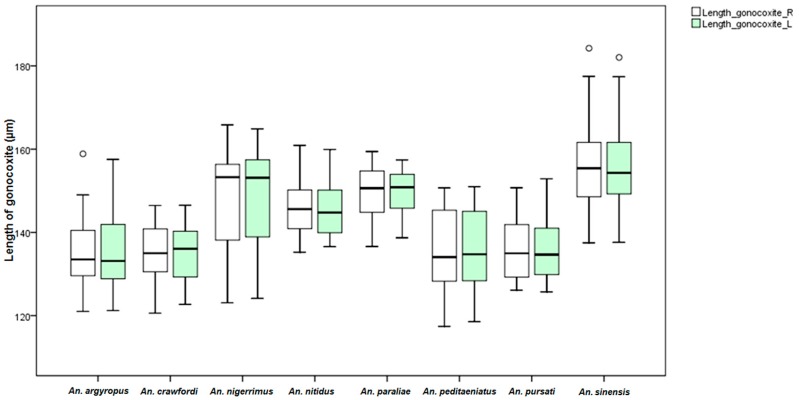
The Box-and-Whisker Plots representation of variation in gonocoxite length of adult male of the eight Hyrcanus Group species.

**Figure 13:**
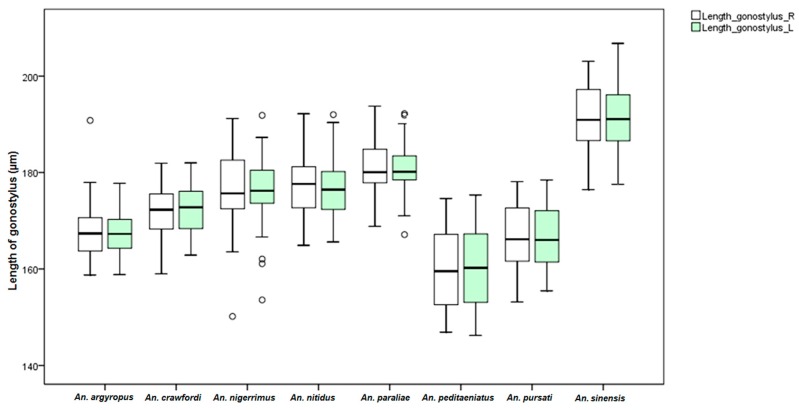
The Box-and-Whisker Plots representation of variation in gonostylus length of adult male of the eight Hyrcanus Group species.

Measurements of male genital structures in the eight species of the Hyrcanus Group are shown in Table 6. The Box-and-Whisker Plots representation of variation in aedeagus length and width, length between base of aedeagus and origin of gonocoxite, width of gonocoxite at origin of parabasal seta, and gonocoxite and gonostylus length of adult male demonstrated that *An*. *sinensis* was longer and wider than other species (Figure 9, Figure 10, Figure 11, Figure 12 and Figure 13).

Comparisons of the results of statistical analyses between stenogamous and eurygamous species was given in Supplementary Material (Table S8). The results show that all measurements of the male genitalia of *An. peditaeniatus* are statistically significant difference from those of the other species (*p* < 0.05). For example, the aedeagus length of *An*. *peditaeniatus* is significantly shorter than the aedeagus of *An*. *argyropus*, *An*. *crawfordi*, *An*. *nitidus* and *An*. *sinensis* (*p* < 0.05). Remarkably, the gonostylus length of *An. peditaeniatus* is significantly shorter than the gonostylus of the seven eurygamous species (*p* < 0.05) (Figure 13).

### 3.7. Clasper Movement and Duration of Mating

The frequency of clasper movement in the stenogamous *An. peditaeniatus* was lower than that in the seven eurygamous species, with statistical significance (*p <* 0.05). Similarly, the mating time of *An. peditaeniatus* was shorter than that in the other species, with statistical significance (*p <* 0.05), except for *An*. *argyropus* (Table 7).

## 4. Discussion

It has long been known that the anopheline mosquitoes have difficulty copulating naturally under laboratory conditions, especially in small spaces, such as a 30 cubic cm cage. However, some species can successfully copulate in small cages, e.g., *Anopheles quadrimaculatus* [35,36], the Gambiae Complex [37], *Anopheles earlei* [38], *An. sinensis* [39,40,41], *Anopheles farauti* [42], *Anopheles albimanus* [43], *Anopheles subpictus* [44], *Anopheles cracens* [29,45], *Anopheles* *annularis* [46], *Anopheles dirus* [47], *Anopheles barberi* [48], *Anopheles sergentii* [49], *Anopheles freeborni* [50], *Anopheles barbirostris* [51], *Anopheles minimus* [52], *Anopheles albitarsis* [53], *Anopheles maculatus* [54], *Anopheles aquasalis* [55], *Anopheles stephensi* [56] and *Anopheles pseudopunctipennis* [57,58]. Therefore, artificial mating techniques have been developed by previous investigators in order to solve the mating problems for maintaining laboratory colonies [59,60].

In view of the success in establishing a stenogamous colony of *An. peditaeniatus*, the possible mechanism that controls its stenogamous behavior was investigated intensively and compared with the behavior of the seven eurygamous species included in this study. The study of stenogamous behavior in adult mosquitoes when mating naturally in 10, 20, 30 and 40 cubic cm cages, with a DRS of 3.6 and 7.2, was carried out using a procedure similar to that detailed by Choochote *et al.* [46], who used a stenogamous colony and DRS of 7.2 for *An*. *annularis*. Three hundred males were deemed appropriate for copulation with 200 females, since male anophelines are monogamous in their mating behavior. In the present study, among the eight species, the highest insemination rates (70–97) were obtained from *An. peditaeniatus* in all cage sizes at both DRS 3.6 and 7.2, whereas *An*. *crawfordi* had the lowest rate (0–4). Remarkably, more than 50% of inseminated females of *An. peditaeniatus* had high sperm density (3+ and 4+) in their spermathecae in all experiments, and no statistically significant difference using various cage sizes and DRS. Thus, it appears that neither cage size nor DRS influenced the mating success of this stenogamous species.

Basically, males within dancing swarms give a mating response when stimulated by flight tones (wing-beat sound) of a conspecific female flying nearby. Sound generated by the beating of female wings consists of a harmonious series that provides most acoustic energy (mostly within the range 200–600 Hz), which is proportional to wing size, wing-beat frequency and ambient temperature [22,23,24,25]. The studies on size assortative mating by Yuval *et al.* [61] demonstrated that a bigger size of male *An*. *freeborni* can mate more often with females than the smaller male. Similarly, Maïga *et al.* [62] found that mated male *Anopheles gambiae* was significantly bigger than non-mated ones. Comparative measurements of wings in adult females and males between the stenogamous and eurygamous species showed that the wings of males were statistically significant difference. *Anopheles* *crawfordi* has the largest size of males, whereas, *An*. *peditaeniatus* is an intermediate-sized male. However, the male size of *An*. *peditaeniatus*, which may involve its mating behavior, corresponds with studies by Ng’habi *et al.* [63], who reported that intermediate-sized males mate more successfully, either due to being more agile in flight or because they can make and maintain contact with females faster and longer within swarms.

The mean palpomere ratios in females were different with statistical significance between stenogamous and eurygamous species. In contrast to this study, Junkum [64] reported no significant difference in the palpomere ratios of adult females between *Anopheles aconitus* Forms B and C. The palpal ratios have been used as taxonomic tools for distinguishing members of the Gambiae Complex, *Anopheles* *melas*, in field studies [65]. Subsequently, Mosha and Mutero [33] reported that the combined values for sensilla coeloconica numbers and palpal ratio could separate only 40.9% of specimens of *Anopheles merus* from freshwater *An*. *gambiae* s.l. It is interesting to note that *An*. *peditaeniatus* was longer in palpomere 4 size than other species, as shown in the Box-and-Whisker Plots. However, certain relationships between the length of palpomere 4 and mating behavior for this stenogamous species are still unclear. 

The olfactory receptor neurons are located in cuticular sensilla on the antennae and maxillary palpi of mosquitoes. Antennae are the major sites of the chemoreceptors that detect and discriminate between air-borne stimuli and guide the females to suitable hosts or to an oviposition site. Hence, it has been assumed that antennal sensilla that most olfaction-driven behaviors, such as host-seeking, oviposition, sources for nectar-feeding, are mediated by these sensilla. Subsequently, other sensory structures on other parts of the body, labellum, tarsi, genitalia, *etc.*, also play an important role in mosquito behavior [66]. The sensory mechanism plays a significant role in host-seeking and oviposition behavior of mosquitoes, which enable them to transmit various diseases to human [67]. Sensilla coeloconica are small, thick-walled sensilla that occur in small and large forms in anophelines [66]. Small sensilla coeloconica have a peg set into the bottom of a pit, but it does not protrude from the opening [66]. These sensilla are of volcano-like structure with an opening at the peak, and they have a much smaller cuticular opening than large coeloconica. Large sensilla coeloconica, commonly called pitted pegs, are absent in the culicines. They appear as round openings in the cuticle, with single peg-shaped setae projecting parallel to the walls of the pit from within. The pegs of large sensilla coeloconica are grooved lengthwise, but with more grooves than sensilla basiconica [34]. This type of sensilla usually locates in a deeply sunken depression of the integument, called a saccalus [68]. The sunken group of sensilla basiconica in both individual sockets and the pit group of the female antennae in *An. barbirostris* also were reported by Kaur [69]. In addition, Ismail [70] and McIver [66] suggested that large sensilla coeloconica are probably olfactory sensilla.

This study is the first to reveal under light microscopy the variation in the number of large sensilla coeloconica on the antennal flagellum of females of the eight species of the Hyrcanus Group. However, it was not possible to count individual sensilla contained within sacculi on the flagellomeres of *An. argyropus* and *An. peditaeniatus*. Interestingly, the cluster of large sensilla coeloconica, which located in sacculi, was found on flagellomeres 3–7 of *An*. *peditaeniatus*. *Anopheles argyropus* also bore a cluster of large sensilla coeloconica on flagellomeres 3–7, whereas the other six species have simple large sensilla coeloconica (pit with a single sensillum) on their flagellomeres instead of clusters. The number of large sensilla coeloconica on female antennae varied from 45/antenna for *An*. *crawfordi* to 89 for *An*. *paraliae*, which is greater than those of *Anopheles maculipennis* (*n* = 28) and *An. stephensi* (*n* = 32) [70,71]. In addition, the mean number of large sensilla coeloconica per antennae of each of the eight species is greater than the number found on the antennae of *An. gambiae* (21.6) and *An. quadriannulatus* (29) [34]. Nevertheless, the comparisons of the mean numbers of these particular sensilla per antennae revealed no statistically significant difference between them. In this study, the cluster of large sensilla coeloconica that are borne on the antennal flagellomeres of females might contribute to the successful mating of the stenogamous *An*. *peditaeniatus* in small cages. 

The behavioral polymorphism, stenogamy/eurygamy, of anophelines has been shown to be inherited and controlled by one or more genes located on the Y-chromosome [72]. Additionally, differences in male genital morphology and frequency of clasper movements have been reported as possibly being involved in the stenogamous behavior of mosquitoes, e.g., stenogamous *An. cracens* and eurygamous *An. dirus* of the Dirus Complex of subgenus *Cellia* [29,45]. The genitalia of *An. cracens* are larger than those of *An. dirus*. This study found differences in the size of the male genitalia of the stenogamous and eurygamous species. The gonostyli of *An*. *peditaeniatus* were significantly shorter than the gonostyli of the eurygamous species, in concert with the findings of Sucharit and Choochote [29]. However, no significance difference was found in size of the gonocoxites. Hence, it might be supposed that a shorter gonostylus could contribute to decrease mating duration (clasping during copulation) for *An*. *peditaeniatus*.

The frequency of clasper movement during induced copulation and mating duration was observed for stenogamous and eurygamous species. The frequency of clasper movement of the stenogamous *An. peditaeniatus* was lower than that in the eurygamous species. Also, the duration of copulation of this species was shorter than that in the other species, except for *An*. *argyropus*. These findings are consistent with those of Sucharit and Choochote [29] who found that the stenogamous *An. cracens* has a lower frequency of clasper movement and shorter period of copulation than *An. dirus*. The shorter duration of pumping motion associated with clasper movement in *Anopheles* *punctipennis* were compared to that of *Anopheles perplexens* [73]. Kanda and Oguma [74] reported that the frequency of clasper movement can be used to distinguish various strains of *An. sinensis* (Japan Strain), which are morphologically highly variable. In addition, Sucharit and Choochote [29] suggested that the morphology of male genitalia and the frequency of clasper movements during induced copulation might be used as a tool for distinguishing *An. cracens* and *An. dirus*.

## 5. Conclusions

Overall, the results of the present study demonstrate that male wing, female maxillary palpomere, characters of the large sensilla coeloconica and gonostylus size might influence the mating success of the stenogamous *An. peditaeniatus*. This study is the first to demonstrate the variation in the number of large sensilla coeloconica on the antennal flagellum of females of eight species of the Hyrcanus Group using light microscopy. However, detailed scanning electron microscopy (SEM) and electrophysiological studies of the different types of sensilla and their distributions on the antennae of females of the eight species of the Hyrcanus Group must be conducted before definite conclusions can be drawn about their function. Furthermore, the lower frequency of clasper movement and shorter mating time could be important mechanisms that control the stenogamous behavior of this species. The structure of the male genitalia of this species influences the success of copulation in a small cage. Therefore, this study contributes knowledge of mosquito biology that may enhance colonization of other anophelines and points to avenues for any future research aspects in Thailand and/or other countries.

## Figures and Tables

**Table 1:** Insemination rates of the eight species of the Hyrcanus Group using various cage sizes (10, 20 and 30 cubic cm cages) at DRS of 3.6.

Mosquito Species	Mean Insemination Rate *		
	10 × 10 × 10 cm **	20 × 20 × 20 cm **	30 × 30 × 30 cm **
*An. peditaeniatus*	88.0	92.0	95.5
*An. argyropus*	44.5 ^a^	49.5 ^a^	65.5 ^a^
*An. crawfordi*	0 ^b^	0 ^b^	4.0 ^b^
*An. nigerrimus*	44.5 ^c^	67.0 ^c^	68.0 ^c^
*An. nitidus*	9.0 ^d^	4.0 ^d^	4.5 ^d^
*An. paraliae*	28.0 ^e^	49.5 ^e^	41.5 ^e^
*An. pursati*	82.0 ^f^	80.0 ^f^	87.5 ^f^
*An. sinensis*	17.0 ^g^	23.0 ^g^	30.0 ^g^

* Dissected 200 spermathecae/species/cage; ** Chi-square test: a, b, c, d, e, f, g *vs.* *An*. *peditaeniatus* (*p* < 0.05).

**Table 2:** Insemination rates of the eight species of the Hyrcanus Group using various cage sizes (10, 20, 30 and 40 cubic cm cages) at DRS of 7.2.

Mosquito Species	Mean Insemination Rate *			
	10 × 10 × 10 cm **	20 × 20 × 20 cm **	30 × 30 × 30 cm **	40 × 40 × 40 cm **
*An. peditaeniatus*	70.0	97.0	78.5	89.0
*An. argyropus*	37.5 ^a^	52.0 ^a^	69.5 ^a^	41.0 ^a^
*An. crawfordi*	0 ^b^	0 ^b^	0.5 ^b^	2.5 ^b^
*An. nigerrimus*	40.5 ^c^	79.0 ^c^	58.0 ^c^	42.0 ^c^
*An. nitidus*	0 ^d^	4.0 ^d^	1.5 ^d^	9.0 ^d^
*An. paraliae*	34.0 ^e^	44.0 ^e^	35.0 ^e^	35.0 ^e^
*An. pursati*	61.0 ^f^	80.0 ^f^	40.5 ^f^	46.5 ^f^
*An. sinensis*	47.5 ^g^	46.0 ^g^	37.0 ^g^	24.0 ^g^

* Dissected 200 spermathecae/species/cage; ** Chi-square test: a, b, c, d, e, f, g *vs.* *An*. *peditaeniatus* (*p* < 0.05).

**Table 3:** Comparisons of mean wing ratios of females and males of the eight species of the Hyrcanus Group (30 females or males/species, *n =* 60).

Mosquito Species	Measurements of Wings of Females (Range)				Measurement of Wings of Males (Range)			
		Length (µm)	Width (µm)	Mean Wing Ratio *		Length (µm)	Width (µm)	Mean Wing Ratio *
*An. peditaeniatus*	Right	1676.89 ± 63.35	462.22 ± 21.15		Right	1594.04 ± 48.17	426.49 ± 12.76	
		(1577.23–1820.49)	(412.21–513.83)	3.63		(1482.64–1728.09)	(402.61–471.53)	3.75
	Left	1677.17 ± 63.75	462.16 ± 20.84		Left	1597.87 ± 48.89	425.99 ± 12.75	
*An. argyropus*	Right	1544.01 ± 83.24	406.94 ± 21.93		Right	1570.52 ± 56.24	393.13 ± 12.98	
		(1281.50–1682.42)	(348.70–449.91)	3.81 ^a^		(1457.86–1683.84)	(372.61–422.80)	4.00 ^h^
	Left	1556.37 ± 85.27	406.78 ± 21.69		Left	1571.88 ± 54.28	393.11 ± 13.17	
		(1285.59–1682.98)	(351.11–449.00)			(1451.24–1681.56)	(370.86–426.61)	
*An. crawfordi*	Right	1757.79 ± 52.51	461.99 ± 15.68		Right	1674.92 ± 70.65	415.68 ± 15.06	
		(1667.70–1863.26)	(431.55–490.29)	3.81 ^b^		(1504.72–1850.72)	(371.58–437.78)	4.04 ^i^
	Left	1757.36 ± 51.72	460.20 ± 14.70		Left	1674.09 ± 70.69	414.86 ± 15.44	
		(1665.63–1856.58)	(430.10–482.59)			(1501.79–1849.61)	(371.01–440.53)	
*An. nigerrimus*	Right	1539.24 ± 69.02	434.00 ± 21.55		Right	1524.94 ± 52.78	384.27 ± 10.81	
		(1377.28–1650.21)	(362.86–470.50)	3.55 ^c^		(1453.34–1683.51)	(354.21–404.76)	3.97 ^j^
	Left	1538.67 ± 68.58	433.75 ± 20.95		Left	1,525.13 ± 54.62	384.86 ± 10.80	
		(1387.92–1650.63)	(363.60–470.50)			(1452.59–1680.61)	(358.71–408.08)	
*An. nitidus*	Right	1539.33 ± 77.41	451.37 ± 23.97		Right	1611.04 ± 45.61	358.95 ± 15.82	
		(1378.83–1676.56)	(381.13–462.01)	3.71 ^d^		(1521.84–1707.28)	(353.13–411.92)	4.17 ^k^
	Left	1539.60 ± 76.95	414.93 ± 22.88		Left	1610.62 ± 45.16	386.06 ± 15.20	
		(1379.23–1675.89)	(381.09–462.63)			(1523.76–1704.20)	(354.54–410.12)	
*An. paraliae*	Right	1716.26 ± 55.43	451.32 ± 18.64		Right	1663.67 ± 48.83	397.85 ± 13.82	
		(1620.99–1825.03)	(409.89–486.01)	3.81 ^e^		(1556.00–1,745.86)	(360.74–425.83)	4.19 ^l^
	Left	1715.10 ± 53.99	450.64 ± 18.73		Left	1662.94 ± 48.19	397.15 ± 13.70	
		(1560.67–1823.87)	(407.68–484.21)			(1559.49–1743.82)	(359.76–422.63)	
		(1577.79–1820.66)	(415.82–512.11)			(1483.24–1729.46)	(401.57–470.30)	
*An. pursati*	Right	1455.64 ± 53.95	398.20 ± 13.85		Right	1424.06 ± 63.55	355.66 ± 17.03	
		(1355.55–1549.94)	(358.12–422.51)	3.66 ^f^		(1248.31–1585.17)	(321.80–389.42)	4.02 ^m^
	Left	1455.30 ± 55.11	398.65 ± 12.97		Left	1424.35 ± 65.77	352.23 ± 23.25	
		(1343.30–1548.63)	(370.92–423.71)			(1254.56–1586.07)	(264.19–388.84)	
*An. sinensis*	Right	1756.11 ± 75.51	468.33 ± 20.49		Right	1626.20 ± 74.16	402.39 ± 17.06	
		(1617.83–1911.86)	(436.35–519.06)	3.75 ^g^		(1453.31–1772.68)	(373.80–434.83)	4.05 ^n^
	Left	1757.11 ± 74.59	468.06 ± 20.37		Left	1626.14 ± 74.02	401.90 ± 17.06	
		(1618.31–1911.33)	(435.02–517.08)			(1456.65–1769.68)	(373.43–433.44)	

* Tukey’s HSD test: a, b, e, g, h, i, j, k, l, m, n *vs.* *An*. *peditaeniatus*, *p* < 0.05; c, d, f *vs.* *An*. *peditaeniatus*, *p* > 0.05.

**Table 4:** Comparisons of the mean palpomeres ratios of females of the eight species of the Hyrcanus Group (30 females/species, *n =* 60).

Mosquito Species	Mean Length of Palpomeres (range)			Mean Palpomeres Ratio * (Range)
	Length of Palpomere 5 (µm)	Length of Palpomere 4 (µm)	Length of Palpomere 3 (µm)	
*An. peditaeniatus*	241.17 ± 15.66 (211.63–263.82)	407.14 ± 23.36 (355.70–445.97)	654.09 ± 33.17 (588.89–730.01)	0.90 ± 0.05 (0.81–1.00)
*An. argyropus*	209.79 ± 23.06 (172.33–253.31)	335.59± 33.86 (259.87–386.26)	525.36 ± 41.03 (434.07–598.88)	1.04 ± 0.08 (0.92–1.27) ^a^
*An. crawfordi*	216.67 ± 20.01 (179.43–279.36)	347.88 ± 27.16 (290.55–402.71)	740.77 ± 32.12 (686.40–812.34)	0.99 ± 0.06 (0.85–1.09) ^b^
*An. nigerrimus*	189.42 ± 17.18 (154.08–224.54)	330.03 ± 27.77 (268.27–388.07)	579.99 ± 29.72 (514.26–640.13)	0.75 ± 0.06 (0.49–0.84) ^c^
*An. nitidus*	201.60 ± 17.30 (174.62–234.18)	329.78 ± 31.12 (248.09–371.48)	593.07 ± 42.18 (507.26–659.56)	0.88 ± 0.05 (0.75–0.96) ^d^
*An. paraliae*	240.92 ± 19.37 (199.52–272.22)	384.22 ± 24.42 (341.02–421.47)	713.43 ± 34.37 (648.87–784.68)	0.86 ± 0.04 (0.77–0.96) ^e^
*An. pursati*	188.99 ± 14.12 (165.65–217.92)	275.09 ± 25.19 (210.15–327.66)	541.04 ± 25.54 (490.78–589.98)	0.90 ± 0.06 (0.77–1.04) ^f^
*An. sinensis*	252.74 ± 72.32 (200.28–551.89)	381.61 ± 38.04 (307.87–488.98)	679.53 ± 87.05 (311.31–771.51)	0.88 ± 0.04 (0.77–0.95) ^g^

* Tukey’s HSD test: a, b, c, d, e, f, g *vs.* *An*. *peditaeniatus*, *p* < 0.05.

**Table 5:** Mean distributions of sensilla coeloconica on the 13 antennal flagellomeres of females of the six species of the Hyrcanus Group (30 females/species, *n =* 60).

Antennal Segment	Mosquito Species							
	CF * (Range)	NG * (Range)	NT * (Range)	PR * (Range)	PS * (Range)	SN * (Range)	PD (Range)	AG (Range)
1	2.35 ± 0.71 (1–4)	3.15 ± 0.69 (2–6)	2.82 ± 0.68 (1–4)	5.20 ± 1.16 (1–7)	1.93 ± 0.52 (1–3)	4.08 ± 0.85 (2–7)	NA	NA
2	3.45 ± 0.87 (2–5)	4.52 ± 1.00 (3–7)	3.67 ± 0.86 (3–6)	6.23 ± 1.03 (4–9)	3.88 ± 0.85 (2–5)	5.12 ± 1.28 (3–8)	NA	NA
3	3.18 ± 0.85 (1–5)	5.17 ± 0.98 (3–7)	4.68 ± 0.70 (3–6)	4.98 ± 1.10 (2–7)	3.80 ± 0.82 (3–6)	5.92 ± 1.18 (3–8)	NA	NA
4	2.42 ± 0.87 (1–5)	4.32 ± 0.97 (2–7)	3.83 ± 0.81 (2–6)	3.65 ± 1.21 (2–6)	4.02 ± 0.95 (2–6)	4.17 ± 0.76 (3–7)	NA	NA
5	2.10 ± 0.63 (1–3)	3.02 ± 0.95 (1–5)	3.08 ± 0.85 (1–5)	2.93 ± 1.01 (1–5)	2.48 ± 0.79 (1–5)	3.22 ± 0.83 (2–5)	NA	NA
6	1.83 ± 0.64 (1–3)	2.68 ± 0.83 (1–4)	2.18 ± 0.73 (1–4)	2.77 ± 0.89 (1–5)	2.57 ± 0.87 (1–5)	2.92 ± 0.79 (1–5)	NA	NA
7	1.70 ± 0.65 (0–3)	2.23 ± 0.81 (1–4)	1.53 ± 0.62 (1–3)	2.37 ± 0.78 (1–4)	1.37 ± 0.64 (0–3)	2.31 ± 0.85 (1–4)	NA	NA
8	1.53 ± 0.60 (1–3)	1.68 ± 0.67 (0–3)	1.23 ± 0.43 (1–2)	1.85 ± 0.80 (1–3)	1.42 ± 0.67 (1–3)	2.10 ± 0.80 (0–4)	NA	NA
9	0.90 ± 0.48 (0–2)	1.03 ± 0.37 (0–2)	0.87 ± 0.39 (0–2)	1.25 ± 0.65 (0–3)	0.98 ± 0.23 (0–2)	0.98 ± 0.43 (0–2)	NA	NA
10	1.02 ± 0.34 (0–2)	0.95 ± 0.39 (0–2)	0.97 ± 0.32 (0–1)	1.07 ± 0.45 (0–2)	1.08 ± 0.38 (0–2)	1.10 ± 0.48 (0–2)	NA	NA
11	1.62 ± 0.72 (1–3)	1.30 ± 0.46 (0–2)	1.28 ± 0.56 (0–2)	1.58 ± 0.59 (0–2)	1.53 ± 0.50 (1–2)	1.45 ± 0.65 (0–3)	NA	NA
12	3.73 ± 1.01 (2–6)	3.02 ± 0.62 (2–5)	2.58 ± 0.53 (2–4)	3.00 ± 0.74 (1–6)	2.75 ± 0.80 (1–5)	3.07 ± 0.80 (1–5)	NA	NA
13	0.02 ± 0.13 (0–1)	0.03 ± 0.18 (0–1)	0.00 ± 0.00 (0)	0.67 ± 0.75 (0–2)	0.03 ± 0.26 (0–1)	0.03 ± 0.18 (0–2)	NA	NA
Total (Range)	25.78 ± 8.48 (45–63)	33.10 ± 8.92 (56–78)	28.73 ± 7.46 (50–69)	37.55 ± 11.15 (52–89)	27.85 ± 8.26 (51–69)	36.47 ± 9.872 (57–88)	NA	NA

CF = *An*. *crawfordi*; NG = *An*. *nigerrimus*; NT = *An*. *nitidus*; PR = *An*. *paraliae*; PS = *An*. *pursati*; SN = *An*. *sinensis*; PD = *An*. *peditaeniatus*; AG = *An*. *argyropus*. NA = not available. * *p >* 0.05, Tukey's HSD test.

**Table 6:** Comparisons of measurements (length and width in microns) of male genital structures in the eight species of the Hyrcanus Group (30 males/species).

Character	Mosquito Species							
	PD (Range)	AG (Range)	CF (Range)	NG (Range)	NT (Range)	PR (Range)	PS (Range)	SN (Range)
Length of aedeagus	37.47 ± 8.14 (25.13–55.60)	40.52 ± 6.39 (30.24–50.28)	52.64 ± 4.66 (40.89–59.56)	46.34 ± 4.30 (38.77–55.34)	42.31 ± 4.93 (35.06–52.13)	50.63 ± 3.43 (43.17–57.41)	40.05 ± 3.65 (35.65–51.95)	57.94 ± 3.60 (50.66–65.47)
Width of aedeagus	33.53 ± 4.16 (25.09–41.43)	36.19 ± 5.43 (26.2–51.29)	37.19 ± 5.37 (26.95–50.60)	36.10 ± 4.22 (28.54–42.63)	33.13 ± 3.60 (26.85–40.95)	32.97 ± 3.34 (28.48–44.58)	30.74 ± 3.38 (24.09–36.11)	37.32 ± 4.38 (31.72–48.53)
Length between base of aedeagus and origin of gonocoxite (right)	47.10 ± 5.77 (38.78–63.36)	44.45 ± 4.98 (35.43–54.93)	49.01 ± 7.68 (31.74–63.86)	44.18 ± 5.89 (31.13–56.32)	45.47 ± 5.09 (31.85–53.73)	45.50 ± 5.47 (31.49–55.91)	45.75 ± 5.89 (38.53–60.95)	52.42 ± 4.18 (43.56–61.78)
Length between base of aedeagus and origin of gonocoxite (left)	47.50 ± 5.01 (39.82–61.77)	44.88 ± 4.70 (36.21–53.94)	48.73 ± 6.87 (32.39–63.10)	44.62 ± 5.29 (31.52–55.32)	45.93 ± 4.94 (31.13–53.69)	45.35 ± 5.37 (33.71–54.20)	45.85 ± 5.80 (39.39–61.68)	52.31 ± 4.14 (44.08–61.87)
Width of gonocoxite at origin of parabasal seta (right)	78.42 ± 7.37 (65.96–92.09)	80.64 ± 4.40 (67.13–88.75)	83.13 ± 3.93 (73.60–90.97)	86.79 ± 5.41 (70.79–95.90)	83.04 ± 5.86 (66.29–91.74)	84.08 ± 3.49 (77.09–89.75)	76.42 ± 6.34 (68.20–94.90)	95.39 ± 5.06 (87.26–105.43)
Width of gonocoxite at origin of parabasal seta (left)	78.60 ± 7.43 (66.26–93.80)	80.27 ± 4.48 (65.99–90.79)	83.22 ± 3.85 (73.80–90.55)	86.07 ± 5.23 (70.48–93.17)	83.96 ± 6.00 (67.69–93.87)	83.74 ± 3.04 (78.29–89.40)	76.25 ± 5.91 (68.30–93.31)	95.10 ± 4.96 (87.19–105.21)
Length of gonocoxite (right)	135.38 ± 9.52 (117.42–150.71)	134.64 ± 8.38 (121.00–158.87)	135.30 ± 6.44 (120.61–146.48)	148.57 ± 11.15 (123.10–165.83)	145.85 ± 6.34 (135.23–160.90)	149.60 ± 6.61 (136.62–159.42)	135.48 ± 6.94 (126.11–150.74)	155.35 ± 9.96 (137.62–182.04)
Length of gonocoxite (left)	135.35 ± 9.30 (118.54–150.99)	135.30 ± 9.10 (121.22–157.54)	135.29 ± 6.14 (122.71–145.53)	149.01 ± 10.79 (124.15–164.87)	145.42 ± 6.14 (136.59–159.93)	149.48 ± 5.92 (138.70–157.41)	135.46 ± 6.85 (125.70–152.87)	191.46 ± 7.08 (176.44–203.08)
Length of gonostylus (right)	160.58 ± 8.85 (146.89–174.59)	167.99 ± 6.41 (158.75–190.81)	172.02 ± 5.71 (159.00–181.93)	175.84 ± 8.18 (150.16–191.19)	177.47 ± 6.75 (164.89–192.20)	180.86 ± 6.21 (168.83–193.76)	167.08 ± 6.65 (153.15–178.10)	191.46 ± 7.08 (176.44–203.08)
Length of gonostylus (left)	160.56 ± 8.84 (146.24–175.32)	167.53 ± 4.44 (158.82–177.75)	172.30 ± 5.35 (162.87–181.99)	176.06 ± 8.02 (153.58–191.86)	177.10 ± 6.59 (165.59–192.01)	180.72 ± 5.78 (167.11–192.24)	166.77 ± 6.28 (155.46–178.45)	191.74 ± 7.21 (177.53–206.80)
Length of gonocoxite + gonostylus (right)	295.96 ± 16.66 (270–322)	302.63 ± 11.36 (285–329)	307.32 ± 10.14 (286–325)	324.41 ± 15.86 (273–352)	323.31 ± 9.89 (303–343)	330.45 ± 10.47 (308–350)	302.56 ± 12.54 (284–326)	346.96 ± 15.36 (315–379)
Length of gonocoxite + gonostylus (left)	295.91 ± 16.42 (268–326)	302.82 ± 10.61 (284–326)	307.59 ± 9.42 (290–324)	325.07 ± 15.52 (278–351)	322.52 ± 9.33 (304–343)	330.20 ± 9.87 (310–348)	302.22 ± 12.07 (286–328)	347.09 ± 15.30 (315–376)

PD = *An. peditaeniatus*; AG = *An. argyropus*; CF = *An. crawfordi*; NG = *An. nigerrimus*; PR = *An. paraliae*; PS = *An. pursati*; SN = *An. sinensis*.

**Table 7:** Comparative measurements of mating time (duration in seconds) and frequency of clasper movement per copulation in the eight species of the Hyrcanus Group (*n* = 30).

Mosquito Species	Frequency of Clasper Movement (Range) *	Mating Time (Range) *
*An. peditaeniatus*	5.62 ± 1.05 (3.5–8.0)	2.9 ± 0.4 (1.5–3.5)
*An. argyropus*	6.68 ± 0.81 (5.5–8.5) ^a^	2.8 ± 0.4 (2.5–4.0) ^h^
*An. crawfordi*	7.83 ± 1.28 (5.0–10.5) ^b^	5.4 ± 0.6 (4.0–7.0) ^i^
*An. paraliae*	8.07 ± 1.03 (6.0–10.0) ^c^	3.9 ± 0.4 (3.5–5.0) ^j^
*An. pursati*	7.22 ± 1.22 (4.5–10.0) ^d^	3.6 ± 0.5 (3–4.5.0) ^k^
*An. nigerrimus*	7.52 ± 1.52 (5.0–11.5) ^e^	4.1 ± 0.8 (2.5–6.0) ^l^
*An. nitidus*	7.87 ± 1.11 (5.5–10.0) ^f^	3.7 ± 0.5 (3.0–5.0) ^m^
*An. sinensis*	8.70 ± 1.40 (7.0–13.0) ^g^	4.6 ± 0.7 (4.0–6.0) ^n^

* Tukey’s HSD test: a, b, c, d, e, f, g, i, j, k, l, m, n *vs.* *An*. *peditaeniatus*, *p* < 0.05; h *vs.* *An*. *peditaeniatus*, *p* > 0.05.

## Supplementary Materials

Supplementary File 1
