# Supplementary material for: Comparative Studies on the Stenogamous and Eurygamous Behavior of Eight Anopheles Species of the Hyrcanus Group (Diptera: Culicidae) in Thailand
Source: Insects. 2016 Mar 26;7(2):11. doi: 10.3390/insects7020011 (PMC4931423; doi:10.3390/insects7020011)
Supplement: Supplementary File 1 [file insects-07-00011-s001.pdf]

insects

MDPI

Supplementary Materials: Comparative Studies on the Stenogamous and Eurygamous Behavior of Eight *Anopheles* Species of the Hyrcanus Group (Diptera: Culicidae) in Thailand

Adulsak Wijit, Kritsana Taai, Watcharatip Dedkhad, Chayanit Hempolchom, Sorawat Thongsahuan, Wichai Srisuka, Yasushi Otsuka, Masako Fukuda and Atiporn Saeung

Table S1. Frequency of inseminated grade based on sperm density in spermathecae of female mosquitoes of the eight species (10 cubic cm cage, DRS = 3.6).

Mosquito Species *	Grading of Sperm Density in Female Spermathecae **			
	1+	2+	3+	4+
An. peditaeniatus	28	30	52	66
An. argyropus	89	0	0	0
An. crawfordi	0	0	0	0
An. nigerrimus	61	24	2[a]	2[a]
An. nitidus	18	0	0	0
An. paraliae	28	4	10[b]	14[b]
An. pursati	114	26	12[c]	12[c]
An. sinensis	12	2	18[d]	2[d]

* Dissected 200 spermathecae/species; ** Chi-square test: a, b, c, d *vs. An. peditaeniatus* ($p < 0.05$).

Table S2. Frequency of inseminated grade based on sperm density in spermathecae of female mosquitoes of the eight species (20 cubic cm cage, DRS = 3.6).

Mosquito Species *	Grading of Sperm Density in Female Spermathecae **			
	1+	2+	3+	4+
An. peditaeniatus	29	23	35	97
An. argyropus	97	2	0	0
An. crawfordi	0	0	0	0
An. nigerrimus	38	56	11[a]	29[a]
An. nitidus	8	0	0	0
An. paraliae	77	10	9[b]	3[b]
An. pursati	93	26	21[c]	20[c]
An. sinensis	10	0	4[d]	32[d]

* Dissected 200 spermathecae/species; ** Chi-square test: a, b, c, d *vs. An. peditaeniatus* ($p < 0.05$).

Table S3. Frequency of inseminated grade based on sperm density in spermathecae of female mosquitoes of the eight species (30 cubic cm cage, DRS = 3.6).

Mosquito Species *	Grading of Sperm Density in Female Spermathecae **			
	1+	2+	3+	4+
An. peditaeniatus	9	37	28	21
An. argyropus	125	6	0	0
An. crawfordi	7	1	0	0
An. nigerrimus	63	60	4[a]	9[a]
An. nitidus	8	1	0	0
An. paraliae	59	13	5[b]	6[b]
An. pursati	89	67	8[c]	11[c]
An. sinensis	25	2	18[d]	15[d]

* Dissected 200 spermathecae/species; ** Chi-square test: a, b, c, d *vs. An. peditaeniatus* ($p < 0.05$).

Table S4. Frequency of inseminated grade based on sperm density in spermathecae of female mosquitoes of the eight species (10 cubic cm cage, DRS = 7.2).

Mosquito Species *	Grading of Sperm Density in Female Spermathecae **			
	1+	2+	3+	4+
An. peditaeniatus	15	5	25	95
An. argyropus	65	10	0	0
An. crawfordi	0	0	0	0
An. nigerrimus	44	14	14 [a]	9 [a]
An. nitidus	0	0	0	0
An. paraliae	52	10	0	4 [b]
An. pursati	100	10	0	12 [c]
An. sinensis	10	10	10 [b]	65 [d]

* Dissected 200 spermathecae/species; ** Chi-square test: a, b, c, d *vs. An. peditaeniatus* ($p < 0.05$).

Table S5. Frequency of inseminated grade based on sperm density in spermathecae of female mosquitoes of the eight species (20 cubic cm cage, DRS = 7.2).

Mosquito Species *	Grading of Sperm Density in Female Spermathecae **			
	1+	2+	3+	4+
An. peditaeniatus	12	4	22	156
An. argyropus	104	0	0	0
An. crawfordi	0	0	0	0
An. nigerrimus	100	4	4[a]	50[a]
An. nitidus	6	1	1[b]	0
An. paraliae	40	6	6[c]	36[b]
An. pursati	92	22	8[d]	38[c]
An. sinensis	62	6	6[e]	18[d]

* Dissected 200 spermathecae/species; ** Chi-square test: a, b, c, d, e *vs. An. peditaeniatus* ($p < 0.05$).

Table S6. Frequency of inseminated grade based on sperm density in spermathecae of female mosquitoes of the eight species (30 cubic cm cage, DRS = 7.2).

Mosquito Species *	Grading of Sperm Density in Female Spermathecae **			
	1+	2+	3+	4+
An. peditaeniatus	12	11	20	114
An. argyropus	138	1	0	0
An. crawfordi	1	0	0	0
An. nigerrimus	66	22	8 [a]	20 [a]
An. nitidus	2	0	1 [b]	0
An. paraliae	36	5	11[c]	18 [b]
An. pursati	68	5	0	8 [c]
An. sinensis	19	6	13 [d]	36 [d]

* Dissected 200 spermathecae/species; ** Chi-square test: a, b, c, d *vs. An. peditaeniatus* ($p < 0.05$).

Table S7. Frequency of inseminated grade based on sperm density in spermathecae of female mosquitoes of the eight species (40 cubic cm cage, DRS = 7.2).

Mosquito Species *	Grading of Sperm Density in Female Spermathecae **			
	1+	2+	3+	4+
An. peditaeniatus	18	38	42	80
An. argyropus	82	0	0	0
An. crawfordi	1	4	0	0
An. nigerrimus	46	24	7[a]	7[a]
An. nitidus	17	1	0	0
An. paraliae	60	0	5[b]	5[b]
An. pursati	85	4	0	4[c]
An. sinensis	44	0	2[c]	2[d]

* Dissected 200 spermathecae/species; ** Chi-square test: a, b, c, d *vs. An. peditaeniatus* ($p < 0.05$).

Table S8. Comparisons of the results of statistical analyses of male genital measurements for the eight species of the Hyrcanus Group.

Character	Mosquito Species		*p*-Value
Length of aedeagus	*An. peditaeniatus vs.*	*An. argyropus*	0.000 *
		An. crawfordi	0.001 *
		An. nigerrimus	0.884
		An. nitidus	0.000 *
		An. paraliae	0.477
		An. pursati	1.000
		An. sinensis	0.000 *
Width of aedeagus	*An. peditaeniatus vs.*	*An. argyropus*	0.252
		An. crawfordi	0.025 *
		An. nigerrimus	0.294
		An. nitidus	1.000
		An. paraliae	1.000
		An. pursati	0.194
		An. sinensis	0.017 *
Length between base of aedeagus and origin of gonocoxite (right)	*An. peditaeniatus vs.*	*An. argyropus*	0.620
		An. crawfordi	0.889
		An. nigerrimus	0.493
		An. nitidus	0.958
		An. paraliae	0.955
		An. pursati	0.984
		An. sinensis	0.009 *
Length between base of aedeagus and origin of gonocoxite (left)	*An. peditaeniatus vs.*	*An. argyropus*	0.546
		An. crawfordi	0.987
		An. nigerrimus	0.419
		An. nitidus	0.962
		An. paraliae	0.733
		An. pursati	0.930
		An. sinensis	0.013 *
Width of gonocoxite at origin of parabasal seta (right)	*An. peditaeniatus vs.*	*An. argyropus*	0.809
		An. crawfordi	0.000 *
		An. nigerrimus	0.000 *
		An. nitidus	0.014 *
		An. paraliae	0.015 *
		An. pursati	0.876
		An. sinensis	0.000 *
Width of gonocoxite at origin of parabasal seta (left)	*An. peditaeniatus vs.*	*An. argyropus*	0.921
		An. crawfordi	0.017 *
		An. nigerrimus	0.000 *
		An. nitidus	0.001 *
		An. paraliae	0.016
		An. pursati	0.668
		An. sinensis	0.000 *

Table S8. *Cont.*

**Character**	**Mosquito Species**		***p*-Value**
Length of gonocoxite (right)	*An. peditaeniatus vs.*	*An. argyropus*	1.000
		An. crawfordi	1.000
		An. nigerrimus	0.000 *
		An. nitidus	0.000 *
		An. paraliae	0.000 *
		An. pursati	1.000
		An. sinensis	0.000 *
Length of gonocoxite (left)	*An. peditaeniatus vs.*	*An. argyropus*	1.000
		An. crawfordi	1.000
		An. nigerrimus	0.000 *
		An. nitidus	0.000 *
		An. paraliae	0.000 *
		An. pursati	1.000
		An. sinensis	0.000 *
Length of gonostylus (right)	*An. peditaeniatus vs.*	*An. argyropus*	0.002 *
		An. crawfordi	0.000 *
		An. nigerrimus	0.000 *
		An. nitidus	0.000 *
		An. paraliae	0.000 *
		An. pursati	0.010 *
		An. sinensis	0.000 *
Length of gonostylus (left)	*An. peditaeniatus vs.*	*An. argyropus*	0.002 *
		An. crawfordi	0.000 *
		An. nigerrimus	0.000 *
		An. nitidus	0.000 *
		An. paraliae	0.000 *
		An. pursati	0.010 *
		An. sinensis	0.000 *
Ratio length and width of gonocoxite	*An. peditaeniatus vs.*	*An. argyropus*	0.646
		An. crawfordi	0.013 *
		An. nigerrimus	1.000
		An. nitidus	0.997
		An. paraliae	0.646
		An. pursati	0.739
		An. sinensis	0.018 *
Ratio length and width of gonocoxite + gonostylus	*An. peditaeniatus vs.*	*An. argyropus*	0.443
		An. crawfordi	0.013 *
		An. nigerrimus	0.000 *
		An. nitidus	0.000 *
		An. paraliae	0.000 *
		An. pursati	0.512
		An. sinensis	0.000 *

* Significantly different ($p < 0.05$, Tukey's HSD test).

© 2016 by the authors; licensee MDPI, Basel, Switzerland. This article is an open access article distributed under the terms and conditions of the Creative Commons by Attribution (CC-BY) license (http://creativecommons.org/licenses/by/4.0/).
